# Expression and Functional Role of Sox9 in Human Epidermal Keratinocytes

**DOI:** 10.1371/journal.pone.0054355

**Published:** 2013-01-18

**Authors:** Ge Shi, Kyung-Cheol Sohn, Zhengjun Li, Dae-Kyoung Choi, Young Min Park, Jin-Hwa Kim, Yi-Ming Fan, Yong Hee Nam, Sooyeon Kim, Myung Im, Young Lee, Young-Joon Seo, Chang Deok Kim, Jeung-Hoon Lee

**Affiliations:** 1 Department of Dermatology, The First Affiliated Hospital of Guangdong Medical College, Zhanjiang, China; 2 Department of Dermatology and Research Institute for Medical Sciences, School of Medicine, Chungnam National University, Daejeon, Korea; 3 Department of Dermatology, College of Medicine, Catholic University of Korea, Seoul, Korea; 4 SkinMED Corporation, Daejeon, Korea; University of Tennessee, United States of America

## Abstract

In this study, we investigated the expression and putative role of Sox9 in epidermal keratinocyte. Immunohistochemical staining showed that Sox9 is predominantly expressed in the basal layer of normal human skin epidermis, and highly expressed in several skin diseases including psoriasis, basal cell carcinoma, keratoacanthoma and squamous cell carcinoma. In calcium-induced keratinocyte differentiation model, the expression of Sox9 was decreased in a time dependent manner. When Sox9 was overexpressed using a recombinant adenovirus, cell growth was enhanced, while the expression of differentiation-related genes such as loricrin and involucrin was markedly decreased. Similarly, when rat skin was intradermally injected with the adenovirus expressing Sox9, the epidermis was thickened with increase of PCNA positive cells, while the epidermal differentiation was decreased. Finally, UVB irradiation induced Sox9 expression in cultured human epidermal keratinocytes, and keratinocytes are protected from UVB-induced apoptosis by Sox9 overexpression. Together, these results suggest that Sox9 is an important regulator of epidermal keratinocytes with putative pro-proliferation and/or pro-survival functions, and may be related to several cutaneous diseases that are characterized by abnormal differentiation and hyperproliferation.

## Introduction

Epidermis, the outermost part of the skin composed mainly of keratinocytes, is a self-renewing, multilayered, stratified and keratinized squamous epithelium. It provides a physical barrier protecting an organism from dehydration and a variety of environmental insults [1]. To generate the protective barrier continuously, keratinocytes should be well balanced in their proliferation, differentiation and apoptosis programs. The mechanism to control keratinocyte differentiation depend on many factors such as calcium, vitamin D, and reactive oxygen species (ROS) [2]–[4]. Many of intracellular signaling cascades and transcription factors are coordinately regulated by these factors, thereby ensuring the proper expression of differentiation-related genes in a spatiotemporal fashion. For example, p63 is expressed in the basal layer of epidermis and supports the proliferation of keratinocytes [5]. In contrast, Notch signaling is activated in spinous layer and induces keratinocyte differentiation [6]–[8]. Also, PKC associated AP1 and C/EBP transcription factors are involved in the induction of keratinocyte differentiation [9]. Although a number of genes required for keratinocyte proliferation and differentiation have been investigated, it is likely that many of important molecules remain to be identified.

Ultraviolet (UV) irradiation is an important physical carcinogen that continuously affects organisms, with the skin the main target. UVB (290–320 nm) is the most effective inducer of sunburn, immediate tanning, and cancers of keratinocyte origin [10], [11]. Although the precise mechanism underlying UVB-induced skin cancers remains to determined, it is regarded that UVB-induced genes in epidermal keratinocytes may have a role in establishing skin cancers.

Sox9 (SRY (sex determining region Y)-box 9) is a transcription factor involved in high mobility group box transcription factor family, and have a role in directing the tissue and cell morphogenesis, survival, and development [12], [13]. Sox9 locus mutations in human can cause campomelic dysplasia which is a skeletal malformation syndrome [14]. In the skin, Sox9 is expressed in the sebaceous gland, sweat gland and outer root sheath of the hair follicles [15]. In addition, Sox9 is highly expressed in the lesion of acne [16]. In mouse, absence of Sox9 in early stem cells can block hair follicle morphogenesis and epidermal wound repair [17], [18].

Although the importance of Sox9 in development is recognized, however, the expression and putative role of Sox9 in epidermal keratinocytes have not been clearly elucidated yet. In this study, we demonstrate that Sox9 is a transcription factor playing an important role in keratinocyte proliferation, differentiation and apoptosis.

## Results

### Expression of Sox9 in Epidermal Keratinocytes

**Figure 1:**
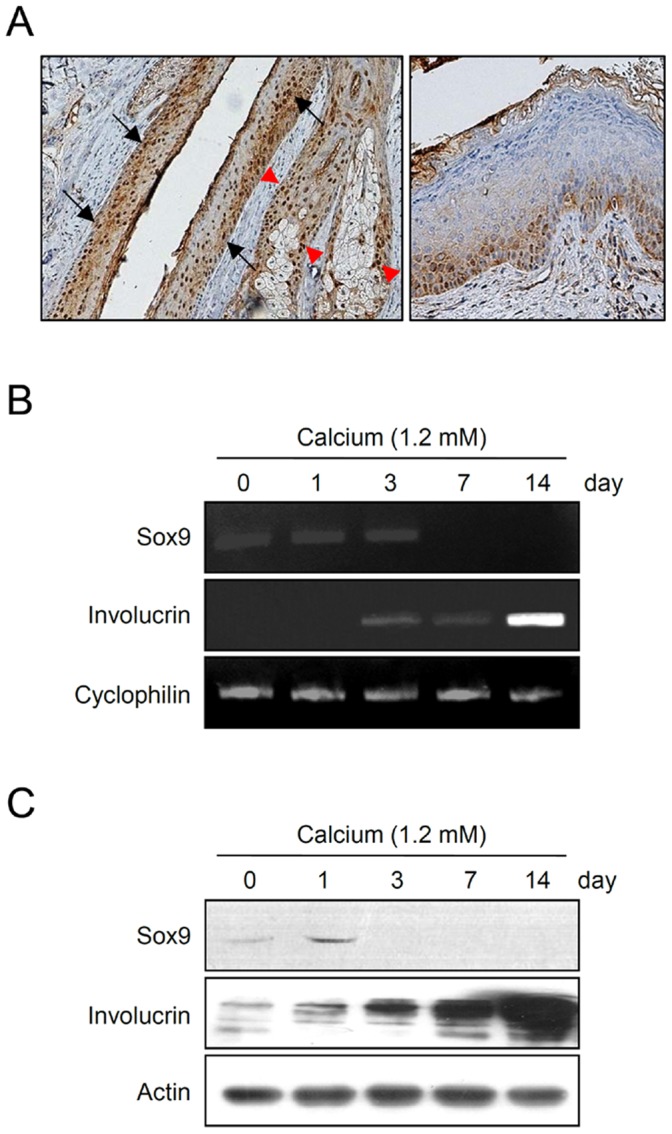
Expression of Sox9 in epidermal keratinocytes. (A) Immunohistochemical staining of Sox9 in scalp skin. Intense nuclear staining is seen in outer root sheath (black arrows) and sebaceous gland (red arrowheads) (left), and interfollicular epidermis (right). (B) Cultured human epidermal keratinocytes were treated with 1.2 mM calcium for the indicated time points. The mRNA level for Sox was decreased in a time-dependent manner during calcium-induced keratinocyte differentiation. Involucrin was used as a positive control for induction of keratinocyte differentiation. Cyclophilin was used as a loading control. (C) The protein level for Sox9 was detected by Western blot analysis. Involucrin and actin were used for positive control and loading control, respectively.

It has been shown that Sox9 is expressed in the outer root sheath of human hair follicle and sebaceous gland [15]. In addition, Sox9 has been detected in normal human undifferentiated epithelial skin cells by in situ hybridization [16]. To confirm the Sox9 expression in epidermal keratinocytes, we first performed immunohistochemistry analysis using human scalp skin. Consistent with previous reports, Sox9 expression was prominently detected in hair follicle outer root sheath and sebaceous gland, with a pattern of higher expression in basal layers of both organs. Similarly, Sox9 expression was also detected in basal layer keratinocytes of interfollicular epidermis (Figure 1A). These results suggest that Sox9 is expressed in undifferentiated rather than in differentiated keratinocytes. To test this idea, we next checked the expression of Sox9 in cultured epidermal keratinocytes, using a well-established calcium-induced differentiation model [19]. RT-PCR analysis showed that expression of Sox9 was decreased after calcium treatment (Figure 1B). Consistent with this result, the protein level for Sox9 was also decreased during the calcium-induced keratinocyte differentiation process (Figure 1C).

### Overexpression of Sox9 Inhibits Keratinocyte Differentiation

**Figure 2:**
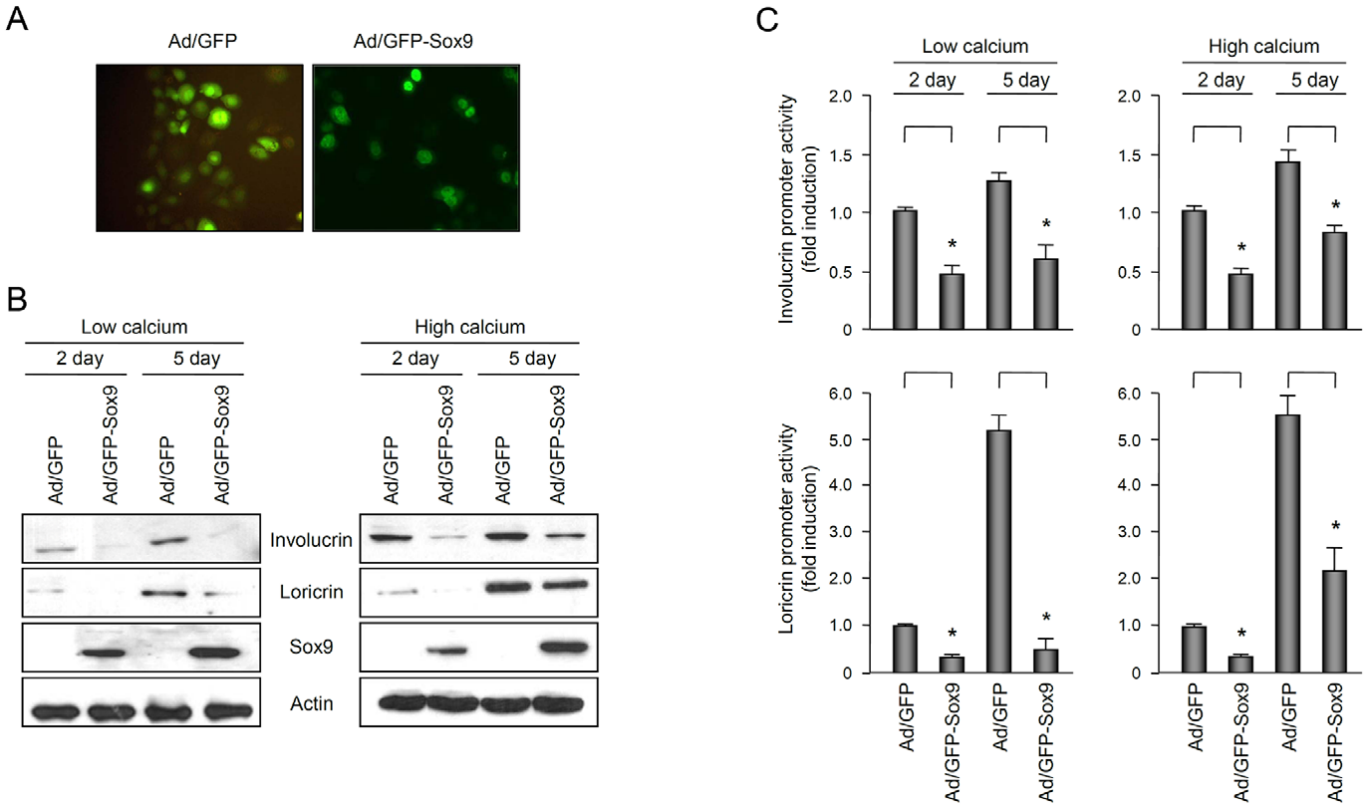
Overexpression of Sox9 inhibits keratinocyte differentiation. (A) Expression of exogenous Sox9 in keratinocytes cultured in vitro. Cells were transduced with adenovirus expressing GFP-tagged Sox9 (Ad/GFP-Sox9) at 10 multiplicity of infection (MOI) for overnight. Cells were replenished with fresh medium and incubated for 2 d. The expression of exogenous Sox9 was observed under the fluorescent microscopy. Adenovirus expressing GFP (Ad/GFP) was used as a negative control. (B) Effect of Sox9 on the expression of differentiation markers. Keratinocytes were transduced with adenovirus expressing GFP-Sox9 at the 10 MOI for overnight. Cells were replenished with fresh medium and incubated for the indicated time points, in a low calcium concentration (<0.1 mM) or a high calcium concentration (1.2 mM). The protein level was verified by Western blot. Involucrin and loricrin are the markers for keratinocyte differentiation. Sox9 bands represent the exogenously expressed GFP-Sox9 fusion protein of ∼82 kDa. (C) The effect of Sox9 on involucrin and loricrin promoter activity. Keratinocytes were transduced with involucrin-luc or loricrin-luc reporter adenovirus together with GFP-Sox9 expressing adenovirus for overnight. Next day, cells were replenished with low calcium (0.01 mM) or high calcium (1.8 mM) medium and incubated for indicated time points. Cells were lysed and assayed for luciferase activity. Data are presented as fold induction and SEM, measured from three independent experiments.

Since the expression of Sox9 was decreased in the differentiated keratinocytes by calcium, we decided to examine whether Sox9 modulates the keratinocyte differentiation. To this end, we constructed a recombinant adenovirus expressing green fluorescent protein-tagged Sox9 (GFP-Sox9), and transduced cultured human epidermal keratinocytes. When overexpressed, Sox9 was located in the nuclei of keratinocytes (Figure 2A). We then determined the effect of Sox9 on the expression of keratinocyte differentiation markers, involucrin and loricrin. Western blot analysis showed that overexpression of Sox9 led to the decrease of involucrin and loricrin protein levels, in both low and high calcium conditions (Figure 2B). Next, we transduced keratinocytes with involucrin-luc and/or loricrin-luc reporter adenoviruses, in which about 3.7 kb of involucrin promoter fragment and 2.0 kb of loricrin promoter fragment were fused to luciferase gene, respectively [20], [21]. Overexpression of Sox9 significantly decreased the involucrin and loricrin promoter activities, irrespective of calcium concentrations (Figure 2C, Figure S1). These results suggest that Sox9 is a functional transcription factor inhibiting keratinocyte differentiation.

### Overexpression of Sox9 Promotes Keratinocyte Proliferation

**Figure 3:**
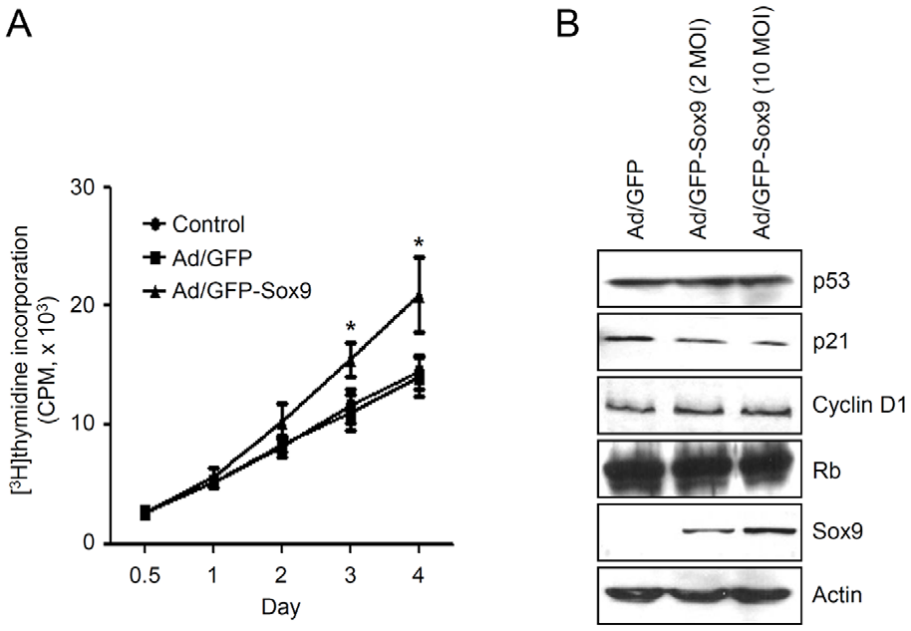
Effect of Sox9 on the growth of keratinocytes. (A) Keratinocytes were transduced with 10 MOI of adenovirus expressing GFP-Sox9 for overnight, washed twice with PBS, and incubated with fresh medium containing [^3^H]thymidine. At the indicated time points, cells were lysed and radioactivity was measured by scintillation counter. Control is non-virus transduced group. (B) Keratinocytes were transduced with adenovirus expressing GFP-Sox9 at the indicated MOI for overnight. Cells were replenished with fresh medium and incubated for 2 d. The protein level for cell cycle modulators were verified by Western blot.

In epidermis, basal layer keratinocytes proliferate and move upwardly, the differentiation process begins in the suprabasal layer and culminates in fully differentiated dead cells on the surface. As the differentiation process takes place along a pathway that leads to cell cycle arrest concomitantly, we next evaluated the effect of Sox9 overexpression on the cell growth. When Sox9 was overexpressed in keratinocytes, significant enhancement of cell growth was observed (Figure 3A). It has been shown that cell cycle modulators, such as p21 and cyclin D1, play roles in keratinocyte proliferation and terminal differentiation [22], [23]. Overexpression of Sox9 resulted in significant decrease of p21, while the cyclin D1, p53 and Rb levels were not affected significantly (Figure 3B, Figure S2).

### Intradermal Injection of Adenovirus Expressing Sox9 Results in Epidermal Thickening and Inhibiting Keratinocyte Differentiation

**Figure 4:**
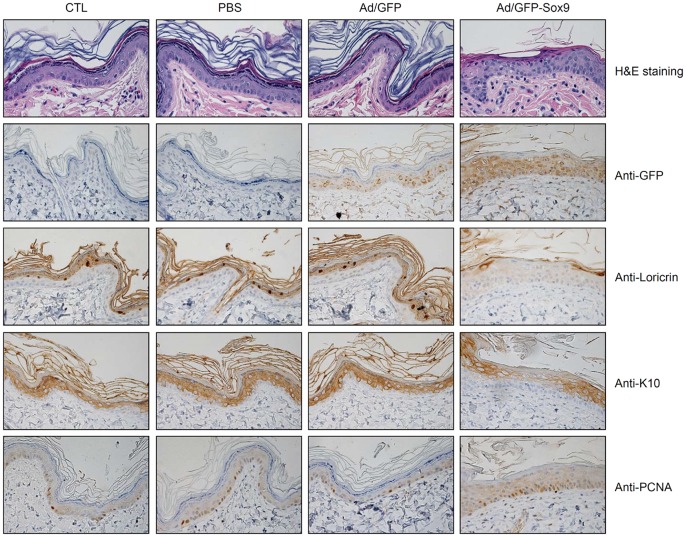
Ectopic expression of Sox9 in rat skin. Female Sprague Dawley (SD) rats were intradermally injected with 50 µl of Sox9 expressing adenovirus (10^9^ particles). After 10 days, skin specimens were harvested and stained with hematoxylin and eosin. Epidermal thickness of Sox9-injected rat is greater than that of GFP-injected rat. CTL, non-injected; PBS, phosphate-buffered saline-injected. Sequentially, paraffin-embedded tissue sections were stained with anti-GFP, anti-loricrin, anti-keratin 10 (K10), and anti-PCNA antibodies. Expression of the early differentiation marker K10 and the late differentiation marker loricrin show that Sox9 can inhibit keratinocyte differentiation. While, ectopic expression of Sox9 increases the cell proliferation in terms of PCNA positivity.

Overexpression of Sox9 in cultured keratinocytes revealed that Sox9 has the potential to induce cell proliferation and to inhibit keratinocyte differentiation. To further confirm the effects of Sox9 in vivo, we performed intradermal injection of purified Sox9 adenovirus into rat skin. Ten days after injection, the thickness of the rat epidermis was markedly increased, compared to the control (uninjected skin) and the PBS or GFP adenovirus injected skins (Figure 4). Consistent with the predominant effects of Sox9 on proliferation and differentiation of cultured keratinocytes, both loricrin and keratin 10 (K10) levels were decreased after intradermal injection of Sox9 adenovirus, and number of PCNA positive cells were increased by Sox9. Together with in vitro and in vivo data, it can be concluded that Sox9 indeed has a functional role during the keratinocyte differentiation.

### Sox9 Protects UVB- Induced Keratinocyte Apoptosis

**Figure 5:**
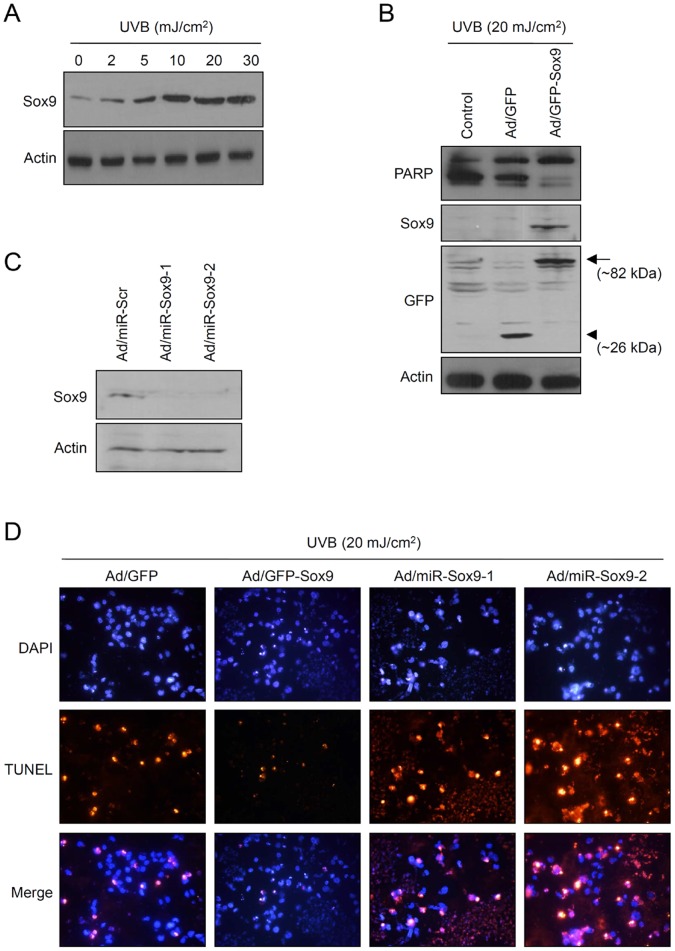
Sox9 protects UVB- induced keratinocyte apoptosis. (A) Keratinocytes were treated with UVB at the indicated doses. Cells were refed with fresh medium and incubated for a further 24 h. Sox9 protein level was increased in a dose-dependent manner. (B) Effect of Sox9 overexpression on UVB-induced keratinocyte apoptosis. Keratinocytes were transduced with 10 MOI of adenovirus for overnight, washed twice with PBS, and incubated with fresh medium for 2 d. Then, cells were UVB-irradiated at the dose of 20 mJ/cm^2^, then further incubated for 24 h. Apoptosis was detected by PARP cleavage. In Sox9 overexpressed group, cleaved PARP (lower band) is significantly reduced, indicating that Sox9 protects UVB-induced keratinocyte apoptosis. In Western blot with anti-Sox9 antibody, a band represents the exogenously expressed GFP-Sox9 fusion protein (∼82 kDa). In Western blot with anti-GFP antibody, upper band (arrow) indicates the GFP-fused Sox9 (∼82 kDa) and lower band (arrow head) indicates the GFP protein (∼26 kDa). (C) Knockdown of Sox9 by microRNA (miR). Keratinocytes were transduced with 10 MOI of adenoviruses expressing miR for Sox9 for overnight. After washing, cells were incubated for a further 2 d, and expression of Sox9 was detected by Western blot. Scrambled (Scr) miR was used for negative control. (D) Keratinocytes were transduced with adenovirus, then UVB-irradiated. Cell apoptosis was analyzed by TUNEL assay. Sox9 overexpression protects UVB-induced apoptosis, while Sox9 knockdown enhances UVB-induced apoptosis.

Skin is the outermost part of organism, and inevitably exposed to various environmental insults. One of most important external insults is ultraviolet (UV) light. Previous studies demonstrate that ultraviolet B (UVB) can induce Sox9 expression in melanocytes and overexpression of Sox9 can increase the pigmentation [24], [25]. We wondered if UVB can induce Sox9 expression in keratinocytes. As expected, when cultured keratinocytes were irradiated with UVB, the expression of Sox9 was increased in a dose dependent manner (Figure 5A). Since UVB is well-recognized apoptosis inducer in keratinocytes, we next investigate whether Sox9 can affect UVB-induced apoptotic process. Keratinocytes were transduced with adenovirus expressing Sox9 and then UVB-irradiated. We then checked the keratinocyte apoptosis by determining PARP cleavage, because a prominent feature of the apoptotic execution phase is the selective cleavage of the nuclear enzyme PARP by caspase-3 [26]. Interestingly, overexpression of Sox9 protected UVB-induced keratinocyte apoptosis in terms of PARP cleavage, compared to control groups (Figure 5B). To further verify the Sox9 effect, we knocked down the expression of Sox9 by transduction of recombinant adenoviruses expressing microRNA (miR) specific for Sox9. Sox9 protein level was efficiently downregulated by adenoviruses expressing miR (Figure 5C). TUNEL assay clearly showed that overexpression of Sox9 protected UVB-induced keratinocyte apoptosis, consistent with PARP cleavage data. In contrast, downregulation of Sox9 by adenoviruses expressing miR led to the increase of UVB-induced apoptosis of keratinocytes (Figure 5D, Figure S3, Table S1).

### Expression of Sox9 in Skin Diseases Related to Keratinocyte Proliferation

**Figure 6:**
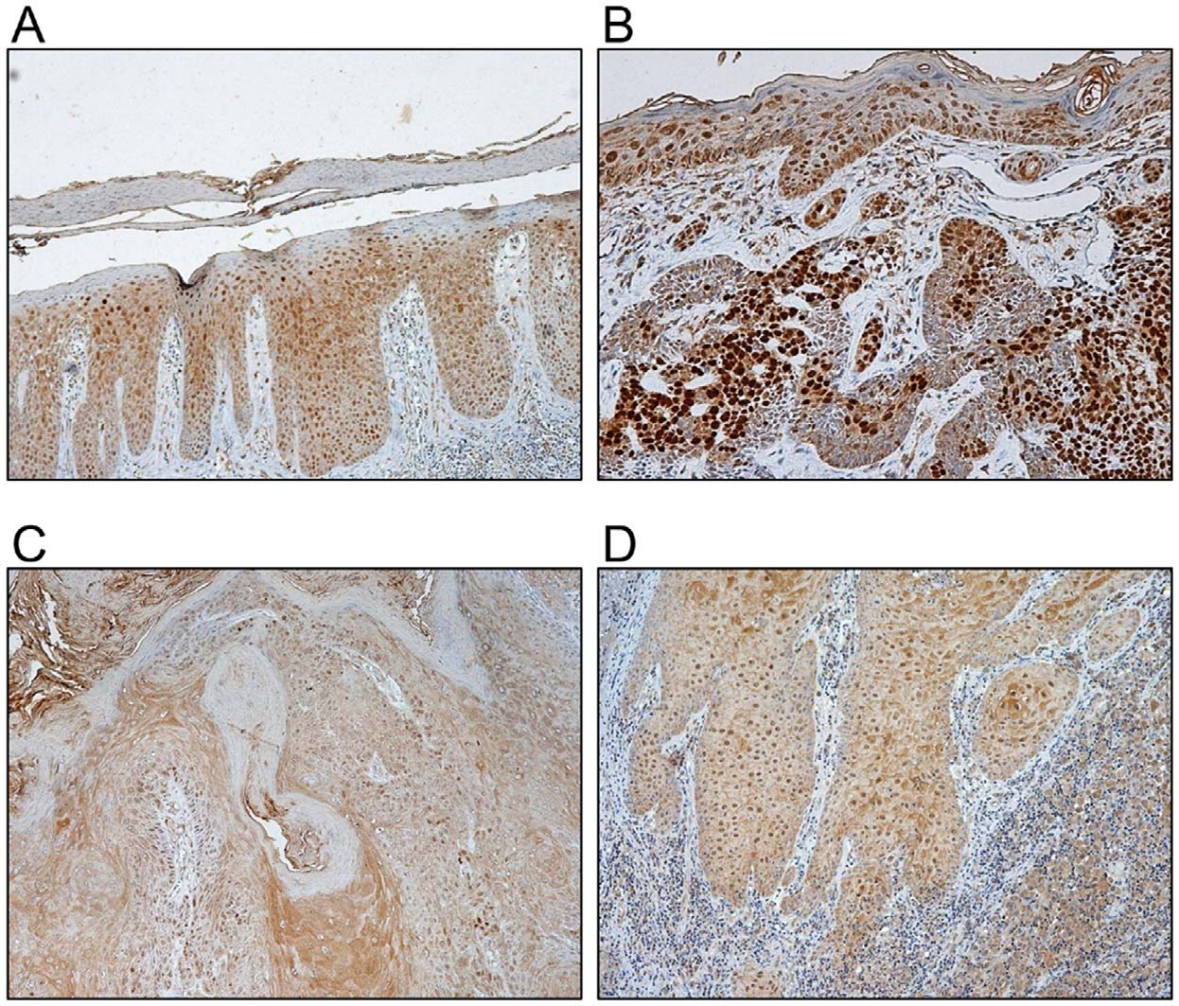
Expression of Sox9 in skin diseases. Paraffin-embedded tissue sections of several skin diseases were stained with anti-Sox9 antibody. (A) psoriasis. (B) basal cell carcinoma (BCC). (C) keratoacanthoma (KA). (D) squamous cell carcinoma (SCC).

Since Sox9 can increase keratinocyte proliferation in vitro and in vivo, we speculated that Sox9 expression may have a correlation to skin diseases relating to cell proliferation. We first determined Sox9 expression in psoriasis, a multisystemic disease characterized by hyperproliferation and altered differentiation of keratinocytes. As a result, intense nuclear staining of Sox9 was observed in both basal and suprabasal layers of psoriatic epidermis (Figure 6A). It has been demonstrated that Sox9 is expressed in all subtypes of basal cell carcinoma (BCC) [15]. Consistent with this finding, in our immunohistochemistry analysis, strong expression of Sox9 was detected in lesion of BCC (Figure 6B). Nuclear staining of Sox9 was also detected in a low-grade skin tumor keratoacanthoma (KA) (Figure 6C), and squamous cell carcinoma (SCC) (Figure 6D).

## Discussion

In this study, we demonstrated that Sox9 is expressed in basal layer of human epidermis, and that Sox9 expression is decreased during calcium-induced keratinocyte differentiation. When Sox9 was overexpressed in both in vitro and in vivo conditions using recombinant adenovirus, cell proliferation was remarkably increased, and keratinocyte differentiation was inhibited. In addition, several skin diseases including psoriasis, BCC, KA and SCC, which are related to disturbance of cell growth control, showed higher expression of Sox9. Based on these results, it can be postulated that the principal task of Sox9 in the epidermis is to control cell proliferation. Keratinocyte differentiation is a process that is regulated by multiple factors including calcium, intracellular signaling cascades and sequential expression of regulatory and structural genes. Besides, one important determinant for keratinocyte differentiation is the cell cycle regulation; that is, differentiation takes place along a pathway towards cell cycle arrest [1]. Thus, the inhibition of keratinocyte differentiation by Sox9 overexpression is likely a secondary event following the increase of cell proliferation, not by a direct regulation of differentiation-related gene expression. This idea was supported by a preliminary experiment in which downregulation of Sox9 by miR failed to increase involucrin level in keratinocytes (Figure S4).

It has been shown that Sox9 expression is regulated by many of upstream signaling pathways in various biological systems. For instance, Wnt signaling pathway promotes chondrocyte differentiation in a Sox9-dependent manner [27], and Sox9 protein is expressed in the intestinal epithelium in a pattern characteristic of Wnt targets [28]. In human outer root sheath cells cultured in vitro, activation of β-catenin pathway increases the Sox9 expression [29]. It has been also suggested that Sox9 is a downstream effector of sonic hedgehog (SHH) signaling pathway, contributing to development of BCC [17]. Furthermore, in melanocytes, UVB upregulates Sox9 expression in cAMP and protein kinase dependent manners [24]. Similarly, in this study, we demonstrated that UVB induces Sox9 expression in epidermal keratinocytes. We also demonstrated that overexpression of Sox9 protects UVB-induced keratinocyte apoptosis. Recently, incidence of skin cancers is gradually growing, and many evidences suggest that exposure to UV radiation is a primary cause of many skin cancers including BCC, KA and SCC [30], [31]. Thus, it is suggestive that Sox9 increase by UVB may be linked to the development of several skin cancers; that is, Sox9 induction may confer the ability of cell survival in harsh condition such as UV irradiation, and Sox9 also play a role in favor of cell proliferation, leading keratinocytes to cancer-prone status. In addition, as previously mentioned, the putative upstream signals for Sox9 include Wnt/β-catenin and SHH pathways, by which several skin cancers such as BCC and SCC can be developed [32], [33], supporting a possible role of Sox9 in skin cancer. The precise relationship and functional roles of Sox9 in establishing skin cancers, however, should be investigated further.

In summary, we demonstrated that Sox9 is an important regulator of keratinocyte proliferation, differentiation and apoptosis. Our data provide clues on possible link between Sox9 and several cutaneous diseases that are characterized by abnormal differentiation and proliferation of keratinocytes.

## Materials and Methods

### Ethics Statement

All human skin samples were obtained under the written informed consent of donors, in accordance with the ethical committee approval process of the Institutional Review Board of Chungnam National University School of Medicine. All animal tests were approved by the Institutional Review Board of Chungnam National University School of Medicine.

### Cell Culture

Primary epidermal keratinocytes were cultured according to the method previously reported [34]. Keratinocytes were maintained in keratinocyte-serum free medium (K-SFM) supplemented with epidermal growth factor (EGF) and bovine pituitary extract (Gibco BRL, Rockville, MD). HEK293 cells were maintained in DMEM medium supplemented with 10% fetal bovine serum (FBS) (Gibco BRL).

### Immunohistochemical Staining

Skin samples were fixed in 10% formalin for 24 h and embedded in paraffin. Sections of skin specimens were dewaxed, rehydrated, then washed three times with phosphate-buffered saline (PBS). After treatment with proteinase K (1 mg/ml) for 5 min at 37°C, sections were treated with H_2_O_2_ for 10 min at room temperature, placed in a blocking-solution (Dako, Carpinteria, CA) for 20 min, followed by reaction with the appropriate primary antibodies. Sections were incubated sequentially with peroxidase-conjugated secondary antibodies (Upstate, Lake Placid, NY) and visualized using a Chemmate Envision Detection Kit (Dako). The following antibodies were used in this study. Sox9 (sc-20095), involucrin (sc-21748), loricrin (sc-51130), GFP (sc-8334), PCNA (sc-7907) and p21 (sc-6246) were purchased from Santa Cruz Biotechnologies (Santa Cruz, CA). Rb (# 9309), p53 (# 9282) and cyclin D1 (#2922) were purchased from Cell Signaling Technology (Danvers, MA). Keratin 10 (PRB-159P) was obtained from Covance (Richmond, CA). Actin (A3853) was purchased from Sigma (St. Louis, MO).

### Reverse Transcription-polymerase Chain Reaction (RT-PCR)

Total RNAs were isolated from keratinocytes using Easy-blue RNA extraction kit (Intron, Daejeon, Korea). Two µg of total RNAs were reverse transcribed with moloney-murine leukaemia virus (M-MLV) reverse transcriptase (ELPIS Biotech, Daejeon, Korea). Aliquots of RT mixture were subjected to PCR cycles with appropriate primer sets. The sequences for primers were as follows: Sox9, 5′- GAGGAAGTCGGTGAAGAACG and 5′-ATCGAAGGTCTCGATGTTGG; involucrin, 5′-CAAAGAACCTGGAGCAGGAG and 5′-CAGGGCTGGTTGAATGTCTT; cyclophilin, 5′-CTCCTTTGAGCTGTTTGCAG and 5′-CACCACATGCTTGCCATCCA. The PCR conditions were as follows: Sox9, annealing temperature 56°C, 32 cycles; involucrin, annealing temperature 56°C, 37 cycles; cyclophilin, annealing temperature 58°C, 24 cycles.

### Western Blot Analysis

Cells were lysed in Proprep solution (Intron). Total protein was measured using a Bradford protein assay kit (Bio-Rad Laboratories, Hercules, CA). Samples were run on SDS-polyacrylamide gels, transferred onto nitrocellulose membranes and incubated with appropriate antibodies. Blots were then incubated with peroxidase-conjugated secondary antibodies, visualized by enhanced chemiluminescence (Intron).

### Adenovirus Creation

An aliquot of RT mixture was subjected to PCR cycles with the primer set for Sox9 (5′-GTACGGATCCATGAATCTCCTGGA and 5′-AATTGCGGCCGCTCAAGGTCGAGT). The amplified full-length cDNA for Sox9 was subcloned into the pENT/CMV-GFP vector that had attL sites for site specific recombination with a Gateway destination vector. Replication-incompetent adenoviruses were created using the Virapower adenovirus expression system (Invitrogen). The adenovirus was purified with cesium chloride according to a method previously reported [35].

For microRNA (miR) specific for Sox9, two sequences were designed that targeted the 3′ untranslated region (UTR) of the human Sox9 mRNA using an Invitrogen’s RNAi Designer. The double-stranded DNA oligonucleotides were synthesized and cloned into the parental vector pcDNA6.2-GW/miR (Invitrogen, Carlsbad, CA). The expression cassette for miR was moved into pENT/CMV vector, and then adenovirus was made in the same way. The miR sequences are as follows: miR-Sox9-1, top strand TGCTGTGTTCTTGCTGGAGCCGTTGAGTTTTGGCCACTGACTGACTCAACGGCCAGCAAGAACA, bottom strand CCTGTGTTCTTGCTGGCCGTTGAGTCAGTCAGTGGCCAAAACTCAACGGCTCCAGCAAGAACAC; miR-Sox9-2, top strand TGCTGTTGACGTGCGGCTTGTTCTTGGTTTTGGCCACTGACTGACCAAGAACACCGCACGTCAA, bottom strand CCTGTTGACGTGCGGTGTTCTTGGTCAGTCAGTGGCCAAAACCAAGAACAAGCCGCACGTCAAC.

### Cell Growth Analysis

For determination of cell growth, [^3^H]thymidine uptake assay was performed. Keratinocytes cells were seeded in 60-mm culture dish, transduced with adenovirus for overnight. Cells were replenished with fresh medium containing 1 µCi of [^3^H]thymidine (Amersham, Buckinghamshire, UK). Following incubation for the indicated time point, cells were washed twice with PBS and incubated with 0.1 N NaOH at room temperature. Radioactivity in cell lysates was measured by liquid scintillation counter.

### Luciferase Assay

Cells were grown at 50% confluency in a 12-well culture plate, then co-transduced with reporter adenovirus and Sox9 expressing adenovirus. After adenoviral transduction, cells were replenished with fresh medium. Cells were further incubated for the indicated time point, and then cellular extracts were prepared using cell lysis buffer. Luciferase activities were determined using Luciferase assay system (Promega, Madison, WI).

### Intradermal Injection of Adenovirus in Rat Skin

Female Sprague Dawley (SD) rats, each weighing approximately 200 g, were used (Orient Bio, Gapyung, Korea). Fifty µl of recombinant virus solution (10^9^ particles) prepared in PBS was injected intradermally into the dorsal skin of rats using a microsyringe with a 28-gauge hypodermic needle. The rats were sacrificed 10 day after intradermal injection and the dorsal skins were removed for histochemical analysis.

### Ultraviolet B (UVB) Irradiation

For UVB irradiation, TL20 lamp (Philips, Eindhoven, The Netherlands) was employed as a light source; this instrument emits the UV light of wavelength ranging between 280 and 320, peaking at 310 nm. Flux intensity was measured by an IL700A research radiometer fitted with an UVB filter and a W diffuser (International Light Inc, Newburyport, MA). UVB irradiation was performed on subconfluent cultures. Control cells were mock irradiated. Before UVB exposure, keratinocytes were washed twice with excess phosphate buffered saline (PBS), and then 1 ml of PBS was added onto 100-mm cell dish during the UVB irradiation. After UVB irradiation, cells were fed with fresh growth medium (0.01 mM calcium). Cells were harvested after 24 h incubation.

### TUNEL Assay

For the direct detection of apoptosis, cells were analyzed by TUNEL kit (Roche Diagnostics GmbH, Mannheim, Germany). Briefly, cells were placed on glass slides and then fixed with 4% paraformaldehyde in PBS for 60 min at room temperature, washed in PBS and permeabilized with 0.1% Triton X-100 in 0.1% sodium citrate for 2 min on ice. TdT labeling was carried according to manufacturer’s instruction.

### Statistical Analysis

Data were evaluated statistically using Student’s t-test. Statistical significance was set at p<0.01.

## Supporting Information

Figure S1 **Keratinocytes were grown at 50% confluency in a 12-well culture plate, then co-transduced with reporter adenovirus (1 MOI) and Sox9 expressing adenovirus at the indicated multiplicity of infections (MOIs).** After adenoviral transduction for overnight, cells were replenished with high calcium (1.8 mM) medium and incubated for a further 2 days. Cellular extracts were prepared using cell lysis buffer and luciferase activities were determined using Luciferase assay system (Promega, Madison, WI). Below the figure, the raw data for involucrin reporter is shown.
(PDF)

Figure S2 **(A) Protein level changes after overexpression of Sox9 were quantified using densitometric analyses of Western blot experiments in** **Figure 3B** **.** The bands were scanned and signals were analyzed by TINA software (version 2.09). Data are average of two independent expreiments. Error bar represents SD. **(B) Another batch of Western blot analysis of Rb protein.**
(PDF)

Figure S3 **Keratinocytes were transduced with 10 MOIs of adenoviruses for overnight, washed twice with PBS, and incubated with fresh medium for 2 d.** Cells were then UVB-irradiated at the dose of 20 mJ/cm^2^, then further incubated for 24 h. Cell apoptosis was analyzed by TUNEL assay. Apoptotic cells were counted and represented as percent of total cells. Data are average of three independent experiments. Error bar represents SD. Statistical significance was set at p<0.05.
(PDF)

Figure S4 **Knockdown of Sox9 by microRNA (miR). Keratinocytes were transduced with 10 MOI of adenoviruses expressing miR for Sox9 for overnight.** After washing, cells were incubated for a further 2 d, and expression of Sox9 was detected by Western blot. Scrambled (Scr) miR was used for negative control. Knockdown of Sox9 does not affect the protein level for involucrin.
(PDF)

Table S1 **Raw data of TUNEL assay.** Keratinocytes were transduced with adenovirus, then UVB-irradiated. Apoptotic cells were counted and represented as percent of total cells.
(XLSX)
