# Supplementary figures and images for: Expression and Functional Role of Sox9 in Human Epidermal Keratinocytes
Source: PLoS One. 2013 Jan 18;8(1):e54355. doi: 10.1371/journal.pone.0054355 (PMC3548846; doi:10.1371/journal.pone.0054355)

Figure S1

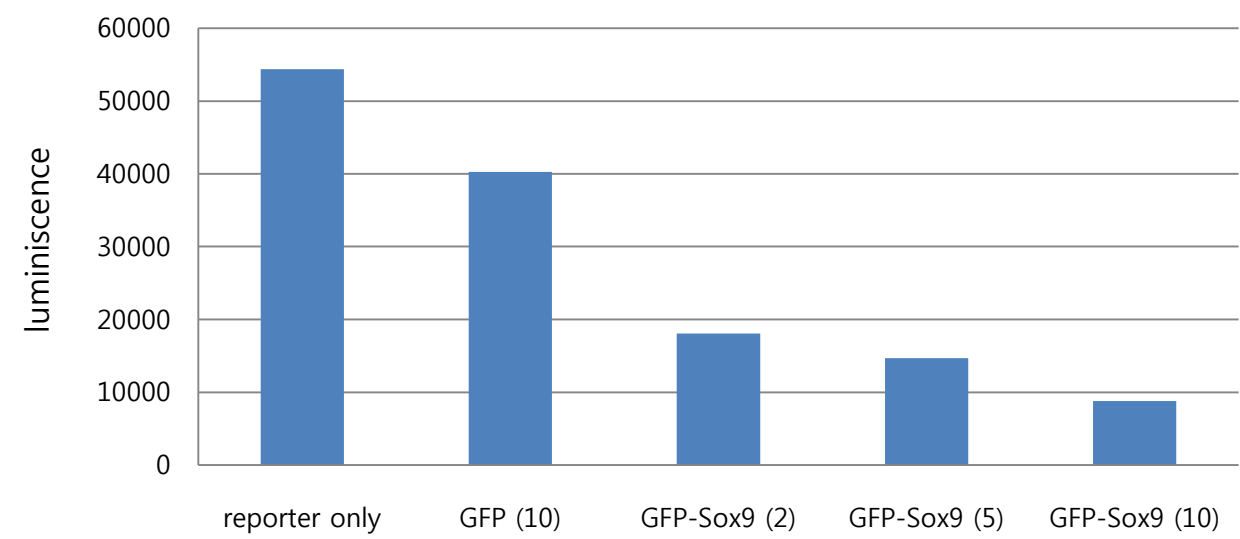

|              |               |          |              |              |               |
|--------------|---------------|----------|--------------|--------------|---------------|
|              | reporter only | GFP (10) | GFP-Sox9 (2) | GFP-Sox9 (5) | GFP-Sox9 (10) |
| luminiscence | 54389         | 40272    | 18070        | 14703        | 8802          |

Supplement: Figure S1 — Keratinocytes were grown at 50% confluency in a 12-well culture plate, then co-transduced with reporter adenovirus (1 MOI) and Sox9 expressing adenovirus at the indicated multiplicity of infections (MOIs). After adenoviral transduction for overnight, cells were replenished with high calcium (1.8 mM) medium and incubated for a further 2 days. Cellular extracts were prepared using cell lysis buffer and luciferase activities were determined using Luciferase assay system (Promega, Madison, WI). Below the figure, the raw data for involucrin reporter is shown. (PDF) [file pone.0054355.s001.pdf]

Figure S2

A

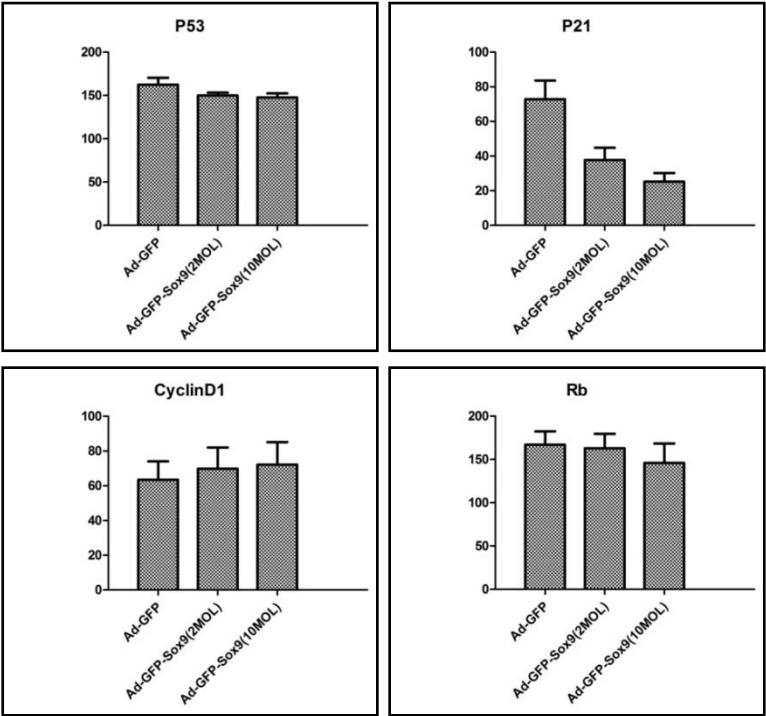

B

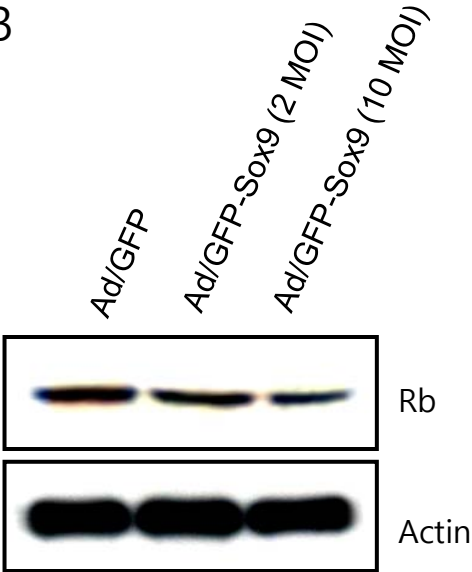

Supplement: Figure S2 — (A) Protein level changes after overexpression of Sox9 were quantified using densitometric analyses of Western blot experiments in Figure 3B . The bands were scanned and signals were analyzed by TINA software (version 2.09). Data are average of two independent expreiments. Error bar represents SD. (B) Another batch of Western blot analysis of Rb protein. (PDF) [file pone.0054355.s002.pdf]

Figure S3

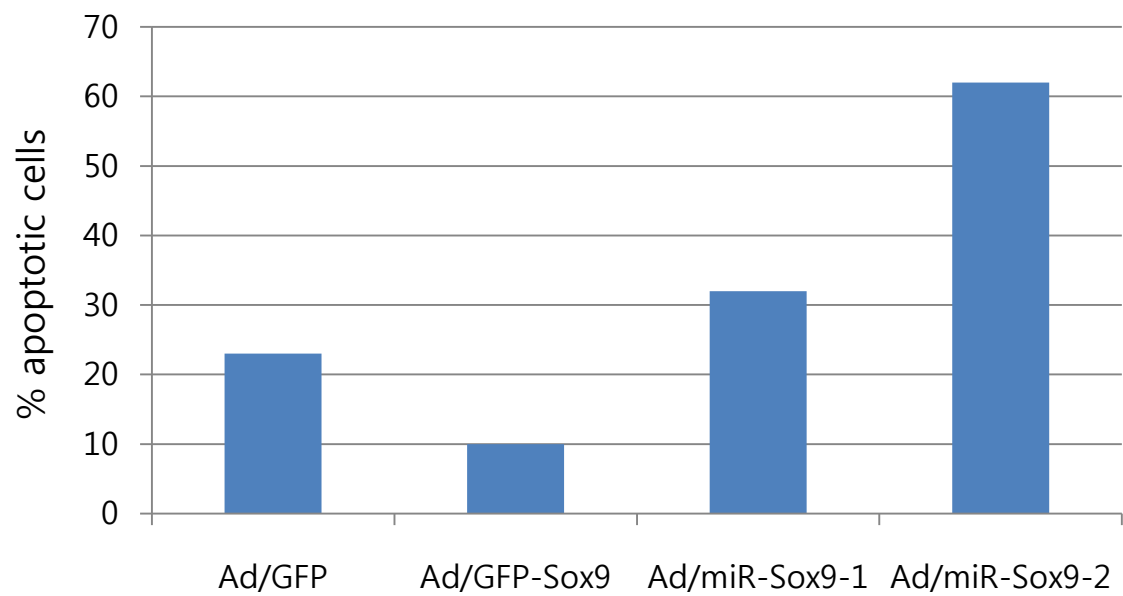

Supplement: Figure S3 — Keratinocytes were transduced with 10 MOIs of adenoviruses for overnight, washed twice with PBS, and incubated with fresh medium for 2 d. Cells were then UVB-irradiated at the dose of 20 mJ/cm2, then further incubated for 24 h. Cell apoptosis was analyzed by TUNEL assay. Apoptotic cells were counted and represented as percent of total cells. Data are average of three independent experiments. Error bar represents SD. Statistical significance was set at p<0.05. (PDF) [file pone.0054355.s003.pdf]

Figure S4

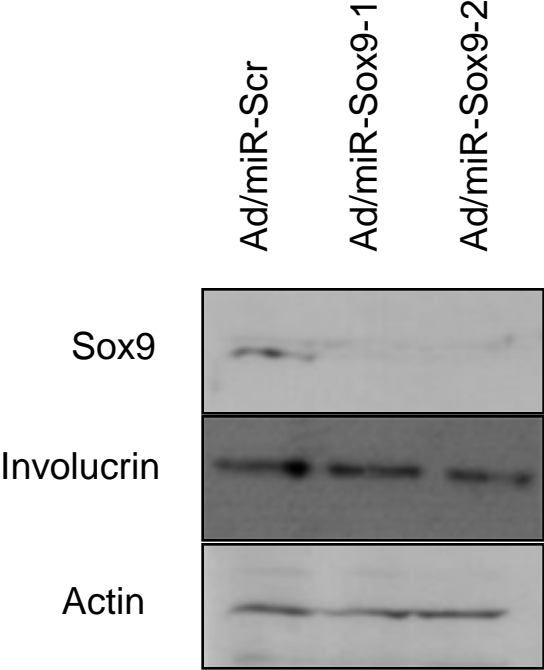

Supplement: Figure S4 — Knockdown of Sox9 by microRNA (miR). Keratinocytes were transduced with 10 MOI of adenoviruses expressing miR for Sox9 for overnight. After washing, cells were incubated for a further 2 d, and expression of Sox9 was detected by Western blot. Scrambled (Scr) miR was used for negative control. Knockdown of Sox9 does not affect the protein level for involucrin. (PDF) [file pone.0054355.s004.pdf]
